# Exercise capacity following a percutaneous endoscopic gastrostomy in a young female with cystic fibrosis: a case report

**DOI:** 10.14814/phy2.12904

**Published:** 2016-08-22

**Authors:** Owen W. Tomlinson, Alan R. Barker, Patrick J. Oades, Craig A. Williams

**Affiliations:** ^1^ Children's Health and Exercise Research CentreSport and Health ScienceUniversity of ExeterExeterU.K; ^2^ Royal Devon and Exeter NHS Foundation Trust HospitalExeterU.K

**Keywords:** Adolescence, exercise testing, respiratory disease, risk factors

## Abstract

Cystic fibrosis (CF) is a genetic condition affecting the respiratory and gastrointestinal systems, with patients experiencing problems maintaining weight, especially during rapid growth periods such as puberty. The aim of this case report was to monitor the effect of gastrostomy insertion and implementation of overnight supplemental feeding upon clinical outcomes, including body mass index (BMI), lung function (FEV_1_), and exercise‐related variables (maximal oxygen uptake [VO_2max_] and ventilatory efficiency [V_E_/VO_2_]) in an 11‐year‐old female with CF. Combined incremental and supramaximal exercise testing to exhaustion was performed at four time points: 3 months prior to the procedure (T1), 2 days prior to (T2), 4 months (T3), and 1 year following the procedure (T4). Improvements following gastrostomy insertion were observed at the 1 year follow‐up with regards to BMI (+20%); whereas absolute VO_2max_ remained stable and lung function fluctuated throughout the period of observation. Declines in function with regards to body weight relative VO_2max_ (−16.3%) and oxygen uptake efficiency (+7.5%) were observed during this period. This case report is the first to consider exercise‐related clinical outcomes in assessing the effect of implementing gastrostomy feeding in CF. The varied direction and magnitude of the associations between variables shows that further investigations are required.

## Introduction

Cystic fibrosis (CF) is a genetically inherited, life‐shortening disease characterized by respiratory and digestive problems which manifests in a decreased exercise capacity (Saynor et al. 2014) and malnutrition (Panagopoulou et al. 2014). Increased mortality risk is reported when patients exhibit decreased lung function and poor nutritional status (Liou et al. 2001). However, exercise‐related predictors of mortality, including maximal oxygen uptake (VO_2max_) (Pianosi et al. 2005a) and peak ventilatory equivalent ratio for oxygen (a measure of ventilatory efficiency; V_E_/VO_2_) (Hulzebos et al. 2014), are also reported in this patient group.

Patients with CF are encouraged to increase their exercise levels (Swisher et al. 2015) and daily caloric intake (Stallings et al. 2008) to improve clinical outcomes, in line with clinical practice guidelines (Cystic Fibrosis Trust, 2011). However, nutritional targets are not always met despite a high level of calorie intake relative to non‐CF controls (Woestenenk et al. 2014). When patients fail to gain weight as predicted and conservative dietary interventions fail, invasive support through the insertion of a percutaneous endoscopic gastrostomy (PEG) may be required. This procedure has been shown to improve nutritional status (Williams et al. 1999) and stabilize lung function (Bradley et al. 2012).

Exercise testing is a valuable tool for evaluating interventions and profiling the clinical status of patients with CF (Cystic Fibrosis Trust, 2011), but has yet to be utilized to assess the effectiveness of this procedure. Therefore, this case report is the first to describe exercise‐related changes alongside nutritional status and lung function following a PEG implant, and supplemental feeding, in a pediatric patient with CF, over a 15‐month period.

## Patient Information

The subject of this case report, an 11‐year‐old female, presented at birth with meconium ileus requiring surgery and was subsequently confirmed to have CF (sweat chloride >60 mmol L^−1^ and homozygous for the ΔF508 mutation).

**Figure 1:**
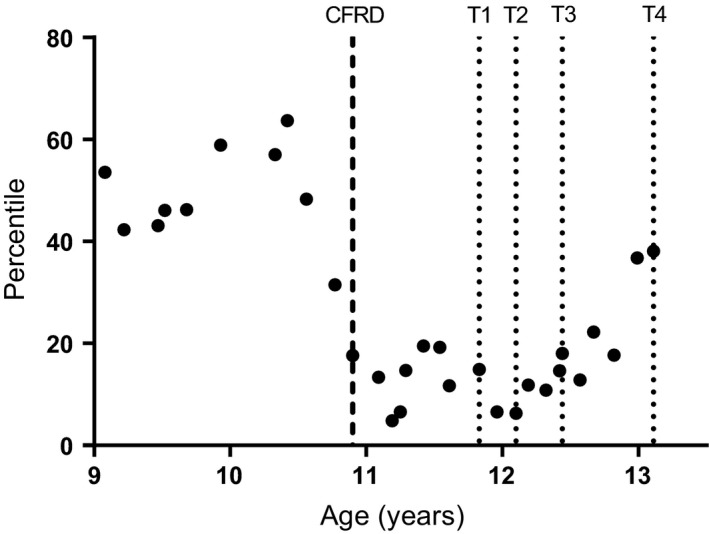
Changes in body mass index as measured by percentile in the 3‐year period preceding the procedure and 1 year following. Dashed line at 10.9 years indicates diagnosis of CFRD. Dotted lines at 11.8, 12.1, 12.4, and 13.1 years indicate T1, T2, T3, and T4, respectively. Percutaneous endoscopic gastrostomy inserted 2 days after T2.

Her clinical course through childhood was complicated by Pseudomonal and Staphylococcal chest infections as well as relapsing Allergic Broncho‐Pulmonary Aspergillosis, treated with recurrent courses of antibiotics, inhaled mucoactives (DNase and hypertonic saline), corticosteroids, antifungals, and chest physiotherapy (autogenic drainage and oscillating PEP). She developed impaired glucose tolerance at age 9, then CF‐related diabetes requiring insulin treatment at 10 years of age (as shown in Fig. 1).

**Figure 2:**
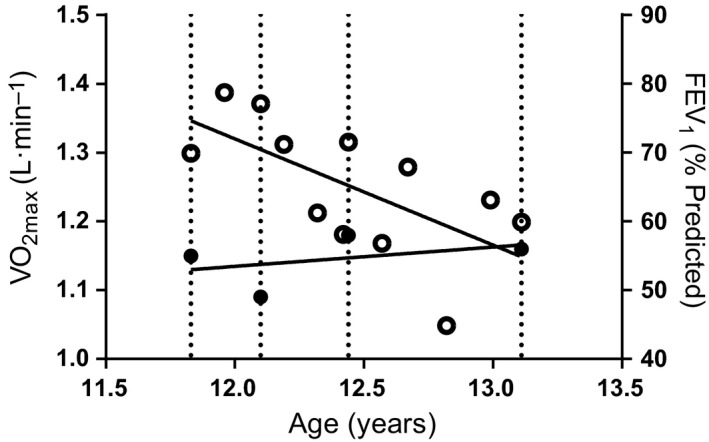
Changes in predicted FEV_1_ (○) (*r *=* *−0.64) and absolute VO_2max_ (●) (*r *=* *0.40) over the 15‐month observation window of this case report. Four vertical lines indicate T1–T4. Percutaneous endoscopic gastrostomy inserted 2 days after T2.

During the 15‐month period reported in this study, the patient was unstable (as shown by FEV_1_ in Fig. 2), and underwent 22 days of intravenous antibiotic treatment.

## Timeline

Changes in clinically important measures of exercise performance over a 15‐month period were assessed for this report, with anthropometric and lung function data provided for the 3 years prior to the procedure and 1 year following. Exercise testing was conducted at scheduled clinical appointments, corresponding to four time points: 3 months preprocedure (T1); 2 days preprocedure (T2); 4 months postprocedure (T3), and 1 year postprocedure (T4).

A fall in body mass index (BMI) from the 64th to 5th percentile in 10 months (Fig. 1) prompted the need to investigate weight gain methods, after conservative dietary changes (visiting the patient's school, meetings with parents, and introduction of twice daily Enshake^®^ drinks) failed. Her growth failed to respond to these noninvasive nutritional supplements, leading to consideration of overnight supplemental feeding via a PEG.

## Diagnostic Assessment

### Anthropometric measures

Height was measured to 0.1 cm (Holtain wall‐mounted stadiometer, Crymych, Wales) and body weight to 0.1 kg (Seca electronic column scale, Birmingham, UK), with BMI compared to normative percentiles (de Onis et al. 2007).

### Lung function

Lung function was assessed using a hand‐held spirometer (MicroPlus, Micro Medical Ltd, Rochester, UK), with maximal (best of three) values of forced expiratory volume in 1 sec (FEV_1_), forced vital capacity (FVC), and FEV_1_/FVC ratio being recorded and compared to normative values (Quanjer et al. 2012).

### Exercise parameters

The patient exercised on an electronically braked cycle ergometer (Lode Excalibur Sport; Lode, Groningen, The Netherlands), completing a validated (Saynor et al. 2013a) combined ramp‐incremental and supramaximal test to exhaustion to determine VO_2max_ and gas exchange threshold (Saynor et al. 2014). Measures of VO_2max_ were normalized to a percentage of predicted maximum (Bongers et al. 2014). The same work rate (15 W·min^−1^), warm‐up, and recovery timings were used across all tests. Pulmonary gas exchange was assessed with a calibrated metabolic cart (Cortex Metalyzer 3B; Cortex Medical, Leipzig, Germany). Blood oxygen saturation (SpO_2_) was measured throughout the test (Nellcor N‐20; Medtronic, Minneapolis, MN) and subjective ratings of perceived effort and dyspnea were assessed on a 1–10 scale.

## Therapeutic Intervention

A PEG tube was inserted under general anesthetic into the stomach, as described previously (Russell et al. 1984). Overnight supplemental feeding with 500 mL of Fresubin^®^ HP Energy (630 kJ/150 kcal) was subsequently introduced. Composition of the feed (per 100 mL) was as follows: 7.5 g protein (20% total energy); 17 g carbohydrate (45%); and 5.8 g fat (35%). This volume avoided interference with morning appetite and minimized vomiting risk with physiotherapy.

## Follow‐Up and Outcomes

### Anthropometric outcomes

Anthropometric and pulmonary outcomes are shown in Table 1. From T1 to T2, increases in height (+1.7 cm), but a fall in body weight (0.6 kg), resulted in a decrease in BMI by 0.63 kg·m^−2^ (−8.6 percentile points). Following the PEG procedure (T3), increases in height (+1.5 cm) and body weight (+3.5 kg), resulted in a gain of 11.7 BMI percentile points (+1.26 kg·m^−2^). At the 1‐year (T4) follow‐up, height had increased by 3.2 cm relative to T2, as had body weight (+9.2 kg) and BMI (+3.02 kg·m^−2^), resulting in an increase to the 38th percentile for BMI.

**Table 1:** Changes in anthropometric and lung function measures over the 15‐month observation period

Variable	T1 (3M‐Pre)	T2 (2D‐Pre)	T3 (4M‐Post)	% Change from T2 to T3	T4 (1Y‐Post)	% Change from T2 to T4
Date	11 June 2014	19 September 2014	19 January 2015		21 September 2015	
Age (years)	11.83	12.10	12.44	2.8	13.11	8.4
Height (cm)	146.6	148.3	149.8	1.0	153.0	3.2
Height (percentile)	29.7	30.2	28.6	−5.3	29.0	−4.0
Weight (kg)	33.9	33.3	36.8	10.5	42.5	27.6
BMI (kg·m^−2^)	15.77	15.14	16.40	8.3	18.16	20.0
BMI (percentile)	14.9	6.3	18.0	185.7	38.1	504.8
FVC (L)	1.67	2.06	2.17	5.3	2.23	8.3
FVC (% predicted)	64.3	76.5	78.3	2.4	75.2	−1.7
FEV_1_ (L)	1.61	1.84	1.76	−4.4	1.58	−14.1
FEV_1_ (% predicted)	69.9	77.1	71.5	−7.3	59.9	−22.3
FEV_1_/FVC (%)	96.41	89.32	81.11	−9.2	70.85	−20.7

Time points: 3M‐Pre (3 months prior to the procedure); 2D‐Pre (2 days prior to the procedure); 4M‐Post (4 months following the procedure); 1Y‐Post (1 year following the procedure). BMI, body mass index; FVC, forced vital capacity; FEV_1_, forced expiratory volume in 1 sec.

### Pulmonary outcomes

Changes in lung function (Fig. 2) showed large variation across the 15‐month observation period, although an overall trend for a decline in function was evident (*r *= −0.64). There was an increase in FEV_1_ from T1 (69.9%) in the lead up to the procedure (T2; 77.1%), before declining at the subsequent observations, T3 (71.5%) and T4 (59.9%).

### Exercise outcomes

Exercise‐related measures are listed in Table 2. Time to exhaustion increased by 49% across all trials (T1–T4), with an 18% increase observed at the 1‐year follow‐up (T2–T4). Absolute VO_2max_ fluctuated over the 15‐month period, decreasing from T1 (1.15 L·min^−1^) to T2 (1.09 L·min^−1^), before increasing at T3 (1.18 L·min^−1^) and T4 (1.15 L·min^−1^). When VO_2_ was expressed relative to body mass a decrease was observed over the 1‐year follow‐up period (−16.3% from T2 to T4), a change associated with the observed weight gain. When normalized for body weight VO_2max_ decreased as a percentage of predicted from 79.3% (T2) to 66.0% (T4).

**Table 2:** Changes in exercise‐related parameters over the 15‐month observation period

Variable	T1 (3M‐Pre)	T2 (2D‐Pre)	T3 (4M‐Post)	% Change from T2 to T3	T4 (1Y‐Post)	% Change from T2 to T4
Peak power (W)	84	98	101	3.1	115	17.4
Exercise duration (min)	4 min 43 sec	5 min 58 sec	6 min 17 sec	5	7 min 3 sec	18
VO_2max_ (L·min^−1^)	1.15	1.09	1.18	8.3	1.16	6.4
VO_2max_ (mL·kg^−1^·min^−1^)	33.8	32.6	32.2	−1.2	27.3	−16.3
VO_2max_ (L·min^−1^; % predicted)	63.8	59.0	62.0	5.1	57.6	−2.4
VO_2max_ (mL·kg^−1^·min^−1^; % predicted)	82.4	79.3	78.1	−1.5	66.0	−16.8
VCO_2_ (L·min^−1^)	1.16	1.25	1.43	14.4	1.50	20.0
RER	1.01	1.15	1.21	5.2	1.29	12.2
V_E_ (L·min^−1^)	40.06	55.78	62.70	12.4	63.80	14.4
V_E/_VO_2_	34.83	51.17	53.14	3.8	55.00	7.5
V_E_/VCO_2_	34.53	44.62	43.85	−1.7	42.53	−4.7
HR_max_ (beats·min^−1^)^a^	196	–	175		–	
GET (L·min^−1^)	0.77	0.71	0.73	2.8	0.65	−8.5
GET (% VO_2max_)	67	65	62	−4.6	56	−13.9
SpO_2_	96	98	94	−4.1	96	−2.0
RPE	5	6	6	0	4	−33.3
RPD	4	3	4	33.3	4	33.3

VO_2max_, maximal oxygen uptake; VCO_2_, maximal carbon dioxide production; RER, respiratory exchange ratio (VCO_2_/VO_2_); V_E_, minute ventilation; V_E_/VO_2_, peak ventilatory equivalent ratio for oxygen; V_E_/VCO_2_, ventilatory equivalent for carbon dioxide; HR_max_, maximal heart rate; GET, gas exchange threshold; SpO_2_, arterial oxygen saturation; RPE, rating of perceived effort; RPD, rating of perceived dyspnea.

a HR_max_ only available for two tests due to equipment malfunction.

Changes were observed in relation to V_E_/VO_2_, with a large increase seen between T1 (34.83) and T2 (51.17). Further, but smaller, increases were then observed at T3 (53.14, +3.8%) and T4 (55.00, +7.5%), relative to T2. V_E_/VCO_2_ decreased over the 1‐year follow‐up (T2 = 44.62, T4 = 42.53; −4.7%), although the magnitude of change was not as large as that of V_E_/VO_2_. Ventilation (V_E_) increased from T2 (55.78 L·min^−1^) to T4 (63.80 L·min^−1^; +14.4%).

## Discussion

This case report shows the inclusion of exercise‐related factors among short‐term fluctuations in clinical measures, following the insertion of a gastrostomy and implementation of overnight feeding in a young CF patient.

For this patient to have been considered a ‘normal’ BMI (i.e., 50th percentile), she was required to weigh 39.75 kg at T2. At T4, this requirement was 44.25 kg. The difference in required and achieved weight at T4 (1.75 kg) relative to T2 (6.45 kg) has justified the requirement of the PEG and supplemental feeding. Such gains are in accordance with prior gastrostomy feeding studies, which have shown similar increases in body weight (Levy et al. 1985) and BMI (Truby et al. 2009).

Although lung function has not been shown to increase following a gastrostomy, studies have shown stabilizing of function (Williams et al. 1999; Bradley et al. 2012). However, these studies only present lung function data at distant time points following such interventions (e.g., 1 year) and do not provide serial measurements, which may bias assessment of intervention efficacy, dependent on the patients function at the time of clinical review. This case reports all clinical visits (averaging every 41 days; range 7–62 days) over the 15‐months follow‐up period and show large fluctuation and a trend for decline in lung function.

As shown in Figure 2, absolute VO_2max_ remained stable over the 15 months, despite the fluctuating FEV_1_, highlighting the independence between the two outcomes. As exercise‐related factors can be predictors of mortality (Pianosi et al. 2005a) and indicators of disease severity (Thin et al. 2002) when they are very low, it is therefore important to incorporate such factors in assessing progression of disease alongside FEV_1_ and BMI.

Changes in absolute VO_2max_ were minimal and fall within the typical error associated with the CPET over the medium term (4–6 weeks; Saynor et al. 2013b) and as such, a minimal change in predicted absolute VO_2max_ at the 1‐year follow‐up (+0.07 L·min^−1^; 59.0–57.6%; Table 2) was observed. However, as VO_2max_ is highly dependent on body size, changes are routinely expressed relative to body weight, thus resulting in a decrease in body weight relative VO_2max_, from T2 to T4 (−5.3 mL·kg^−1^·min^−1^; 79.3–66.0% predicted; Table 2). This change is greater than previously observed annualized declines (Pianosi et al. 2005b) and the decline in predicted relative VO_2max_ has a greater magnitude of change than the predicted absolute VO_2max_ value. This decline is of particular relevance given its clinical implication (i.e., risk of mortality (Pianosi et al. 2005a) and hospitalization (Pérez et al. 2014)). Although it would normally suggest a deconditioning effect, it could be proposed that the rapid increase in weight (+9.2 kg from T2 to T4, resulting in an increase of 31.8 BMI percentile points) is driving this change and deconditioning is not in fact occurring. However, to appropriately determine and interpret such changes, an accurate assessment of body composition (e.g., skin folds) is required. However, clinical constraints prevented such measures in the current report.

Increases in V_E_ without a corresponding increase in VO_2max_ (Table 2) indicate a reduction in the efficiency of gaseous exchange. However, given the increase in VCO_2_ alongside the increase in V_E_ and the stability of V_E_/VCO_2_, it can be suggested that an increase in CO_2_ release may be driving the change in ventilation. This is supported by the rise in RER from T1 to T4, suggesting an increased ‘non‐metabolic’ increase in CO_2_ at maximal exercise perhaps due to increased anaerobic metabolism, carbohydrate metabolism, and/or CO_2_ storage during exercise. The increase in peak power (exercise performance) indicates an increase in muscle power, but as no increase in absolute VO_2max_ was observed, this suggests that oxidative capacity of the muscle was not enhanced and a greater contribution likely originated from anaerobic metabolism.

## Conclusion

This case report has provided novel data combining clinical and exercise measures in a young patient with CF following the implementation of gastrostomy feeding. Of the key measures described, BMI increased, whereas relative VO_2max_ showed a decline due to body weight changes, amid a fluctuating FEV_1_. Furthermore, absolute VO_2max_ remained stable against a decreased function of V_E_/VO_2_. The direct impact of the feeding protocol upon exercise capacity cannot be directly obtained due to the patients’ clinical instability and lack of a control patient. This case report does highlight the utility of exercise and body composition testing in assessing the outcome profile of individual patients following interventions, warranting its further use in the assessment and treatment of CF.

## Informed Consent

The patient upon whom this report is focused was previously involved in a NHS Regional Ethics Committee approved study, examining integrated approaches to exercise in CF. Observations made in this report are part of the study follow‐up, to which patient assented and parent have consented to release of data.

## Conflict of Interest

None declared.
